# The second of a series of articles for the 60^th^ anniversary of the Brazilian Society of Genetics

**DOI:** 10.1590/1678-4685-GMB-2016-00040002

**Published:** 2016

**Authors:** Francisco Mauro Salzano, Fabrício Rodrigues dos Santos, Klaus Hartfelder, Carlos F.M. Menck

**Affiliations:** 1 Departamento de Genética, Instituto de Biociências, Universidade Federal do Rio Grande do Sul, Porto Alegre, RS, Brazil.; 2 Departamento de Biologia Geral, Instituto de Ciências Biológicas, Universidade Federal de Minas Gerais, Belo Horizonte, MG, Brazil.; 3 Departamento de Biologia Celular e Molecular e Bioagentes Patogênicos, Faculdade de Medicina de Ribeirão Preto, Universidade de São Paulo, Ribeirão Preto, SP, Brazil.; 4 Departamento de Microbiologia, Instituto de Ciências Biomédicas, Universidade de São Paulo, São Paulo, SP, Brazil.

This issue of GMB (39.4) brings the second series of articles especially written and dedicated to the 60^th^ anniversary of the Brazilian Society of Genetics (SBG). This series includes three reviews, two research articles and a short communication. Dr. Azevedo and his group present an interesting discussion on the interaction of endophytic bacteria with the phytopathogen *Xylella fastidiosa*. The work discusses this interaction and proposes the potential use of these endophytic bacteria as an interesting strategy for the biological control of citrus variegated chlorosis (CVC), the disease caused by *Xylella fastidiosa* in orange plantations. Dr. Guerra presents a critical review on the role of agmatoploidy and other forms of chromosomal rearrangements in plant karyotype evolution. He emphasizes that , although the concept of agmatoploidy was proposed more than 60 years ago, there are, in fact, only few examples where chromosomal duplication is generated by this process. Dr. Dietrich and her colleague Dr. Dragatsis present an overview on Familial Dysautonomy (FD), a major genetic disorder within the Hereditary Sensory and Autonomic Neuropathies. They discuss the epidemiology of FD, as well as the main clinical symptoms. FD is caused by a point mutation in the *IKBKAP* gene that encodes a protein involved in multiple intracellular processes, including neurtotrophic retrograde transport. Specific functional aspects of this gene can now be studied in a mouse model. Dr. Hutz and her group discuss the different origins of sickle cell disease caused by mutations in the beta globin gene (HBB*S gene). They present evidence supporting that these mutations were introduced into the American continent basically by gene flow from Africa during the slave trade from the 16th to the 19th century. Considering the world population, four haplotypes predominate, including Bantu (CAR), Benin (BEN), Cameroon (CAM) and Arabian-Indian (ARAB), indicating the effects of old migration events in the dispersion of this disease. However, the authors also emphasize that recent internal migration waves may be responsible for the increase in the frequency of sickle cell disease in the Brazilian population. Working with plants, Dr. Margis-Pinheiro and her group investigate the diversity and evolution of the diacylglycerol acyltransferase (*DGAT*) gene. They propose that the four paralogous genes found in plants may have distinct origins, and that studying their evolution and expression may provide important information on their roles in plant cells. Finally, Dr. Schneider and his group present molecular data indicating the hybridization between different species of squirrel monkeys (of the genus *Saimiri*, Cebidae). Curiously, they present data that support the fact that such monkeys can still keep fertility.

We hope you enjoy these high quality scientific articles and the other excellent works published in this issue.

Fabrício Rodrigues dos Santos, Francisco Mauro Salzano, Carlos FM Menck and Klaus
Hartfelder FRS and FMS are Guest Editors of the SBG 60 years Special Series of
Articles and CFMM and KH Editors of Genetics and Molecular Biology

